# Anticonvulsant Therapy in Trigeminal Neuralgia: A Class-Oriented Systematic Review

**DOI:** 10.3390/medicines13010003

**Published:** 2026-01-26

**Authors:** Miguel Pinto Moreira, Bruno Daniel Carneiro, Carlos Silva Faria, Daniel Humberto Pozza, Sara Fonseca

**Affiliations:** 1 Experimental Biology Unit, Department of Biomedicine, Faculty of Medicine of Porto, University of Porto, 4200-319 Porto, Portugal; miguel.pintomoreirapm@gmail.com (M.P.M.); bcarneiro@med.up.pt (B.D.C.); 2 Rheumatology Service, Unidade Local de Saúde do Alto Minho, Hospital Conde de Bertiandos, 4990-078 Ponte de Lima, Portugal; 3 Department of Surgery and Physiology, Faculty of Medicine of Porto, University of Porto, 4200-319 Porto, Portugal; carlosafaria@gmail.com (C.S.F.); saracunhafonseca@gmail.com (S.F.); 4 Institute for Research and Innovation in Health and IBMC, University of Porto, 4200-135 Porto, Portugal; 5 Anesthesiology Department, São João University Hospital Centre, 4200-135 Porto, Portugal

**Keywords:** anticonvulsants, sodium channel blockers, gabapentin, levetiracetam, pharmacological therapy, neuropathic pain, facial pain disorder, trigeminal pain, carbamazepine

## Abstract

Background/Objectives: Trigeminal Neuralgia (TN) is a chronic neuropathic condition characterized by sudden, severe facial pain. Anticonvulsants are the cornerstone of pharmacological management, yet comparative evidence based on pharmacological class remains scarce. This systematic review aimed to evaluate the efficacy and safety of anticonvulsants in TN, stratified by their mechanism of action. Methods: A systematic search in PubMed, Scopus and Web of Science was conducted following PRISMA 2020 guidelines. Studies employing a pharmacological approach including human patients with TN, published in English since 2000, were included. Risk of bias was assessed using the Cochrane RoB 2, the ROBINS-I and the ROBINS-E tools, according to the study design. Results: Out of 922 initial records, 12 studies met the eligibility criteria. Sodium channel inhibitors showed high efficacy but frequent adverse effects, particularly hyponatremia and central nervous system symptoms. Calcium channel modulators offered a more favorable safety profile. Combination therapies showed benefits, levetiracetam and topiramate were moderately effective and well tolerated. Although the evidence has limitations, anticonvulsants continue to be the primary treatment for TN. Sodium-channel blockers demonstrate strong efficacy, whereas alternative agents generally provide superior tolerability. Conclusions: These findings support selecting drugs according to their underlying mechanisms of action. Equally important is tailoring therapy to pain phenotype and patient characteristics, balancing mechanism with tolerability and efficacy.

## 1. Introduction

Trigeminal neuralgia (TN) is a chronic neuropathic condition marked by sudden, severe, stabbing episodes of facial pain within the distribution of one or more trigeminal nerve branches, usually occurring unilaterally [1]. The trigeminal nerve consists of three main branches: the ophthalmic (V1), maxillary (V2), and mandibular (V3) divisions. The first two are purely sensory, whereas the mandibular branch also carries motor fibers. Primary sensory input converges on the trigeminal (Gasserian) ganglion in Meckel’s cave, before projecting to the spinal trigeminal nucleus, the ventral posteromedial thalamic nucleus, and ultimately the primary somatosensory cortex [2].

This disorder most commonly affects the maxillary and mandibular branches, with bilateral involvement being rare. Approximately 36–42% of patients present with V2 and V3 involvement [2]. The average age of onset is between 50 and 60 years, with increasing incidence among elderly populations. Prevalence estimates range from 12 to 27 cases per 100,000 people, and the condition disproportionately affects women [3,4]. Pain episodes are brief, lasting seconds to minutes, and are typically described as electric shock-like or stabbing. Benign stimuli, such as chewing, speaking, or light touch, may trigger attacks, and patients often develop anticipatory anxiety between paroxysms [3]. The disease significantly impairs quality of life (QoL), contributing to functional disability, sleep disturbance, and psychological comorbidities such as depression and anxiety [4,5].

According to the International Headache Society (IHS) classification, trigeminal neuralgia (TN) is divided into three subtypes: classic TN, which results from neurovascular compression; secondary TN, attributable to multiple sclerosis (MS), tumors, or other structural lesions; and idiopathic TN, in which no clear etiology can be identified [3,6]. While vascular compression leading to focal demyelination of trigeminal root entry zone is the most widely accepted mechanism, recent studies suggest the involvement of abnormal sodium channel expression, central sensitization, and impaired inhibitory neurotransmission as additional pathophysiological contributors [7,8].

Pharmacological management remains the cornerstone of therapy. The IHS recommends anticonvulsants as first-line agents, with carbamazepine (CBZ) and oxcarbazepine (OXC) being the most widely prescribed [3]. CBZ provides initial pain relief in approximately 75% of patients, but treatment response may diminish over time, and side effects, such as dizziness, somnolence, and hyponatremia, often limit tolerability [3]. OXC demonstrates efficacy comparable to carbamazepine (CBZ) with a better safety profile, but its tolerability advantage is partly offset by a higher risk of hyponatremia among its adverse effects. Gabapentin (GBP) and pregabalin are being used more frequently, especially for patients with coexisting neuropathic pain conditions. Evidence indicating that GBP may exceed CBZ in efficacy, tolerability, and safety highlights the importance of reconsidering existing first-line treatment recommendations [5,9].

Adjunctive and second-line therapies are under active investigation, including intravenous phenytoin and lacosamide for acute exacerbations [10], botulinum toxin type A (BoNT-A) injections with high response rates [11,12], and novel agents such as eslicarbazepine, lamotrigine and the selective Nav1.7 blocker vixotrigine [13,14]. Intravenous lidocaine, ketamine infusions, cannabinoids, and calcitonin gene-related peptide (CGRP) monoclonal antibodies [9,15], although not the standard of care, highlight a shift toward multimodal and mechanism-based pain management strategies.

Surgical interventions are considered when pharmacological management is not sufficient or adverse effects become intolerable. Microvascular decompression provides superior long-term outcomes compared with percutaneous rhizotomies or stereotactic radiosurgery, particularly in patients with classic TN and confirmed vascular compression [3,16]. However, surgery carries inherent procedural risks and is not suitable for all patients

Despite the widespread clinical use of anticonvulsants, the literature remains fragmented, with reviews often focusing on single drugs rather than drug classes or shared mechanisms of action, including multimodal/multidisciplinary approach. To our knowledge, no systematic review has yet synthesized the evidence comparing anticonvulsants by pharmacological class. Addressing this gap is essential to guide clinical decision-making and optimize personalized treatment strategies for TN.

Although anticonvulsants are widely used to treat TN, the evidence base is fragmented: most reviews examine single agents in isolation rather than grouping drugs by shared pharmacology or mechanism of action [5,12,17]. A class-oriented synthesis is important because drugs that act on the same target may show consistent patterns in efficacy, dose–response, adverse-effect profiles, and interactions that are obscured when each drug is considered separately. Unlike prior single-drug reviews, a pharmacological-class approach allows pooled assessment of class-level benefits and harms, better handles heterogeneity across small trials, and highlights mechanistic rationales for choosing one class over another in specific clinical contexts. To our knowledge, no systematic review has yet compared anticonvulsants at the class level.

This systematic review addresses that gap and aims to systematically evaluate the efficacy of anticonvulsants used in the treatment of trigeminal neuralgia, grouped by pharmacological mechanism of action.

## 2. Materials and Methods

A systematic review was undertaken following the Preferred Reported Items for Systematic Reviews and Meta-Analyses (PRISMA) guidelines [18,19].

### 2.1. PICO Question

“In patients with trigeminal neuralgia (P), how does the efficacy of different classes of anticonvulsant drugs (I) compare with each other (C) in reducing pain intensity and the frequency of pain episodes (O)?”

### 2.2. Eligibility Criteria

Inclusion criteria: Studies written in English, performed on adult human patients, including randomized clinical trials (RCTs), interventional and non-interventional observational studies, and case series with at least 5 participants, provided they included one of the two pharmacological treatment options.

Exclusion criteria: Studies that were conducted on animals, other reviews, case reports with fewer than 5 participants, editorials, and gray literature or conference abstracts were excluded by design. Additionally, studies published prior to the year 2000 were not considered for inclusion in this review.

### 2.3. Search Strategy

The study protocol was registered in PROSPERO (International Prospective Register of Systematic Reviews) under the code CRD42024599049 on 19 October 2024. The original aim was to evaluate all pharmacologic options used as alternatives or adjuncts to anticonvulsants in trigeminal neuralgia (TN).

A search was performed at Scopus, PubMed, and Web of Science databases. The search strategy combined free-text terms and controlled vocabulary (MeSH/keywords) tailored to each database. For PubMed, we used the MeSH-based queries (“Trigeminal Neuralgia” [Mesh] AND “Anticonvulsants” [Mesh]). In Scopus, the search string included (“Trigeminal Neuralgia” AND “Anticonvulsants”). For Web of Science, the topic-based queries (“Trigeminal Neuralgia” AND “Anticonvulsants”) were applied.

Duplicate records were first identified and excluded prior to screening. Subsequently, two independent researchers conducted an initial assessment of titles and abstracts to determine study eligibility during October and November 2024.

### 2.4. Manuscript Selection

Manuscripts were selected in the Rayyan platform (https://www.rayyan.ai/ accessed on 20 October 2024) with “blind mode” activated for the rigor of the process. Conflicts were solved with a meeting in December 2024, for a careful analysis and discussion to reach an agreement. Full-text articles were subsequently reviewed to determine eligibility for inclusion. Furthermore, the included manuscripts were carefully analyzed to ensure appropriate inclusion. A third researcher helped to resolve any doubts.

The studies were systematically assessed to include only those with suitable methodologies and relevant outcomes for the present manuscript, allowing the results validity and reliability. A third researcher reviewed all the information. In cases of missing information or unavailable full texts, the corresponding authors were approached for clarification or access. The level of agreement was assessed using the Kappa test [20].

Eligible studies were reviewed, and data were manually extracted by two authors and compiled into a structured table for analysis in the months of January 2025 and May of 2025.

For each included study, we extracted the following data: first author, year of publication, country, study design, sample size, patient characteristics (including age and sex), pharmacological treatments evaluated, and all predefined outcomes. Primary outcomes focused on pain reduction, typically measured using validated pain scales or responder criteria (e.g., significant decreases in pain intensity or frequency). Secondary outcomes encompassed functional improvement, quality-of-life and emotional measures, time to treatment response, the need for additional therapies, and the occurrence of adverse effects.

### 2.5. Risk of Bias Assessment

The risk of bias for each included study was independently assessed by two authors using tools appropriate to the study design. For randomized controlled trials (RCTs), the revised Cochrane Risk of Bias tool (RoB 2) [21] was used, which evaluates bias across five domains: (1) the randomization process, (2) deviations from intended interventions, (3) missing outcome data, (4) measurement of the outcome, and (5) selection of the reported result. Each domain is rated as “low risk”, “some concerns”, or “high risk”, leading to an overall judgment of bias for the study. For non-randomized studies, either the Risk Of Bias In Non-randomized Studies—of Exposures (ROBINS-E) [22] or the Risk Of Bias In Non-randomized Studies—of Interventions (ROBINS-I) [23] was applied, depending on whether the study examined exposures or interventions. These tools assess bias across multiple domains, including confounding, participant selection, classification of exposures/interventions, deviations from intended interventions, missing data, outcome measurement, and selective reporting. Each domain is rated from “low” to “critical” risk of bias, which informs the overall study-level judgment. Final assessments were visualized and summarized using the Risk-of-bias VISualization tool (robvis) to facilitate transparent comparison across studies [24].

## 3. Results

Our initial search returned 922 articles: 208 from Web of Science, 246 from PubMed and 468 from Scopus. After removing duplicate records, 625 entries remained. Based on title and abstract, records were screened and 612 were excluded. The remaining 13 reports were assessed for eligibility. One study was excluded because the study population consisted of patients with facial neuropathic pain of diverse etiologies rather than trigeminal neuralgia.

**Figure 1:**
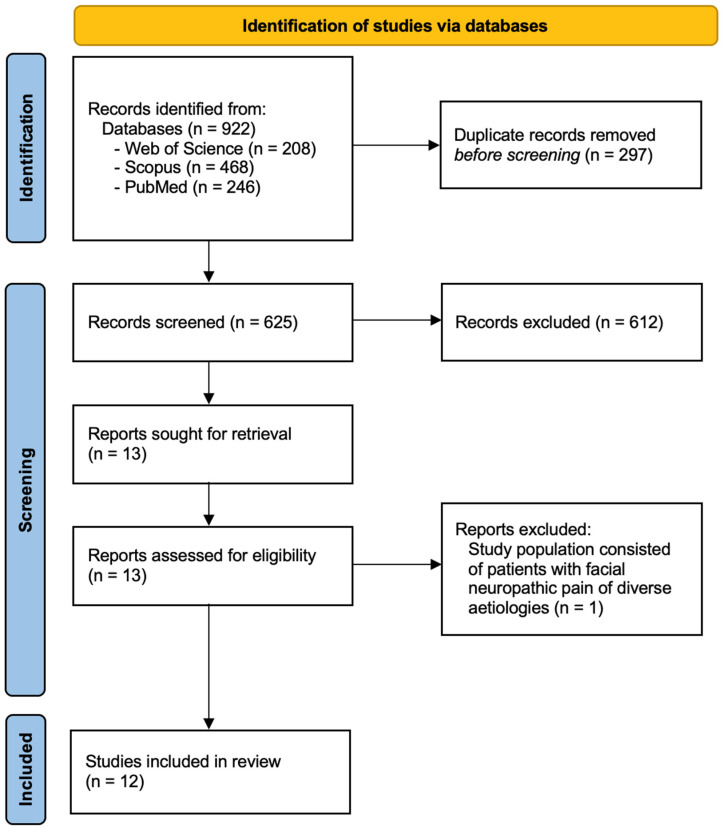
PRISMA flowchart of literature search, study screening, and inclusion.

Twelve studies were finally included in this review and the flow diagram of the selection process is depicted in Figure 1. The Kappa test agreement between the authors was 0.93. Disagreement was resolved by consensus among four of the five authors.

**Figure 2:**
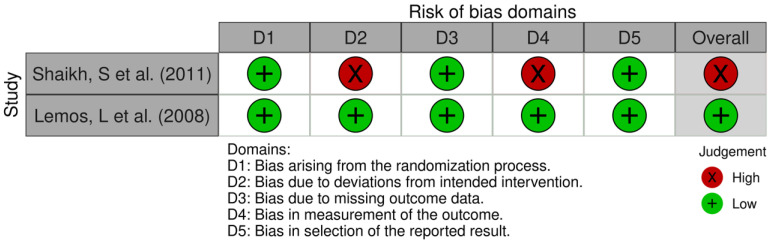
Risk of bias domains assessment of randomized trials according to Cochrane risk of bias tool [25,26].

**Figure 3:**
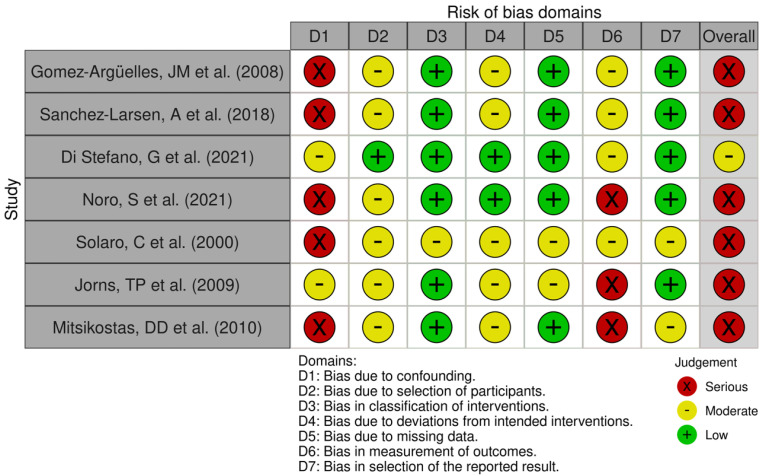
Assessment of Risk of Bias Domains in Non-Randomized Studies According to the Risk of Bias in Non-Randomized Studies of Interventions (ROBINS-I) [27,28,29,30,31,32,33].

**Figure 4:**
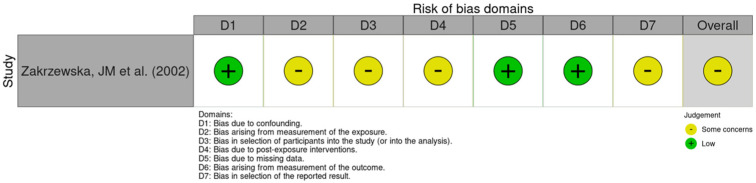
Assessment of Risk of Bias Domains in Non-Randomized Studies According to the Risk of Bias in Non-Randomized Studies of Exposures (ROBINS-E) [34].

Two RCT [25,26] were assessed for bias using RoB 2 (Figure 2), with an overall risk of bias rated as “high” in one of them and “low” in the other. Non-randomized studies were evaluated using ROBINS-I (Figure 3) or ROBINS-E (Figure 4), depending on whether they investigated an intervention or an exposure. Seven studies [27,28,29,30,31,32,33] were assessed using ROBINS-I, with an overall risk of bias rated as “serious” in six of them and “moderate” in the remaining one. One study [34] was evaluated using ROBINS-E, with an overall risk of bias rated as “some concerns”. Two studies—a case series by Solaro et al. (2018) [35] and a case series by Domingues et al. (2007) [36]—were not assessed using these tools, as their design is not compatible with the risk-of-bias frameworks applied to comparative or analytical studies. The obtained results are shown in Figure 2, Figure 3 and Figure 4 (PRISMA checklist is provided in the Supplementary File).

### 3.1. Description of Included Studies

The extracted data characteristics for each study are available in Table 1. The summary of key findings involving the main pharmacological approach for TN is depicted in Table 2. The 12 studies included in this review assessed drug responsiveness by reporting the number of patients who improved, based on reductions in pain intensity and frequency, improvements in quality of life or emotional variables, and evaluated tolerability through the documentation of adverse effects. The following main results extracted from the studies were divided according to the pharmacological class under study. The first 6 articles correspond to sodium channel inhibitors. The 7th article refers to calcium channel inhibitors. Articles 8 and 9 combine these two mechanisms of action. Articles 10 and 11 refer to the pharmacological action on the synaptic vesicle protein 2A (SV2A). The 12th article is related to topiramate, which primarily acts on glutamate receptor α-amino-3-hydroxy-5-methyl-4-isoxazolepropionic acid (AMPA).

### 3.2. Sodium Channel Inhibitors

Sodium channel inhibitors remain central to TN management, with carbamazepine (CBZ) and oxcarbazepine (OXC) showing comparable efficacy across studies. A large retrospective analysis demonstrated similar pain control for CBZ and OXC, although both were limited by CNS adverse effects and hyponatremia, particularly in older patients and those with idiopathic or secondary TN [29]. Hyponatremia was reported in nearly one-third of OXC users, but rarely required treatment discontinuation [27].

OXC is also effective in CBZ-resistant cases, although its long-term benefit may be limited, as many refractory patients eventually required surgery despite short-term response [34]. This supports OXC as a useful option in resistant TN while emphasizing earlier consideration of surgical management in highly selected cases.

Newer agents offer additional alternatives. Lamotrigine provides pain control comparable to CBZ with fewer hematologic and CNS side effects, suggesting value for intolerant patients [25]. Eslicarbazepine acetate appears effective and well tolerated, including in multiple sclerosis-associated TN, though hyponatremia monitoring remains important [28]. Intravenous fosphenytoin offers rapid rescue therapy for acute attacks while awaiting surgical treatment [30]. Overall, sodium channel inhibitors remain effective first-line options, with treatment choice largely guided by tolerability, patient age, and TN subtype.

### 3.3. Calcium Channel Inhibitors

Gabapentin (GBP), a calcium channel α2δ ligand, has demonstrated efficacy in idiopathic TN, particularly when combined with local anesthetic blocks. Combination therapy can provide faster and greater pain relief, improved quality of life, and was well tolerated, with no reported adverse effects. These findings suggest gabapentin, especially in combination with local blocks, may be a viable alternative for patients unable to tolerate carbamazepine, though larger trials are needed to confirm efficacy and evaluate variability in response [26].

### 3.4. Both Sodium and Calcium Channel Inhibitors

Combination therapy targeting both sodium and calcium channels may provide synergistic benefit in TN, particularly in patients with multiple sclerosis (MS). CBZ plus gabapentin and lamotrigine plus gabapentin both produced significant pain relief while reducing side-effect burden compared to high-dose monotherapy, without detectable pharmacokinetic interactions [31]. Similarly, it was demonstrated in CBZ-refractory MS patients that low-dose lamotrigine combined with pregabalin was well tolerated and provided meaningful analgesia [35]. These findings support the potential of rational polytherapy (multimodal) to optimize efficacy while minimizing adverse effects, though confirmation in larger cohorts is required.

### 3.5. Synaptic Vesicle SV2A

Levetiracetam, titrated up to 3, 4 or 5 g/day, has shown limited but meaningful pain relief in some TN patients. It is generally well tolerated with promising efficacy and safety, supporting its potential role as an adjunct in complex or refractory cases [32,33].

### 3.6. Topiramate

Topiramate, at low doses (50–100 mg/day), provided early and sustained pain relief with good tolerability in most patients with classic or symptomatic TN, including those previously treated with anticonvulsants. These results suggest topiramate may be a useful alternative for patient’s intolerant to first-line therapies [36].

### 3.7. Additional Laboratory Findings

Oxcarbazepine was reported as having a favorable safety profile, with no significant hematological abnormalities and minimal biochemical effects, aside from an impact on sodium levels [27,34]. Its relative safety is further supported by findings of only a few cases of elevated liver enzymes and a single instance of thrombocytopenia [29]. In contrast, CBZ is associated with a higher incidence of adverse effects, including liver enzyme elevations, anemia, leukopenia, and thrombocytopenia, particularly at higher doses [25,29]. LMT also presents some hematological, hepatic, and renal abnormalities, though less frequently than CBZ [25]. Plasma levels of GBP, CBZ, and LMT remained within normal ranges in a small patient sample [31], while levetiracetam showed no hematological or biochemical effects and did not interfere with other anticonvulsant levels [32].

## 4. Discussion

This review confirms that anticonvulsants, particularly sodium channel blockers, remain the foundation of trigeminal neuralgia management, while growing evidence supports the integration of additional therapies selected based on efficacy, tolerability, and mechanism of action. The twelve included studies investigated diverse pharmacologic classes, including sodium channel blockers, calcium channel modulators, synaptic vesicle protein 2A (SV2A) modulators, and glutamate receptor antagonists. These drug classes represent distinct strategies aimed at correcting the neurophysiological mechanisms underlying TN, specifically aberrant neuronal excitability and dysfunctional pain signaling. By organizing therapies according to their molecular targets, this review offers a structured synthesis of the evidence and underscores class-specific differences in both clinical effectiveness and adverse effect profiles.

Sodium channel blockers, mainly CBZ and OXC, remain the most widely studied and commonly prescribed options. The studies in this review confirm their efficacy, particularly in classical TN, with initial response rates above 80% in some cohorts [29,34]. Tolerability, however, is a major limitation: CBZ is frequently associated with central nervous system adverse effects, hyponatremia, and hematological disturbances [25,29]. OXC appears somewhat better tolerated but carries a considerable risk of hyponatremia, especially in elderly populations [27,29,34]. These real-world observations align with registry data documenting similar efficacy and side-effect frequencies [29] and are consistent with international guidelines that still endorse CBZ as first-line therapy, with OXC as an alternative when side effects or contraindications arise [37]. Nevertheless, long-term symptom control often falters, with many patients needing dose escalation, polytherapy, or surgical referral. Importantly, longer disease duration may negatively impact surgical outcomes [34,37], underscoring the need for timely diagnosis, early optimization of pharmacotherapy, and consideration of individualized treatment strategies to improve long-term prognosis. Additionally, recent cadaveric work showing neural tension patterns linking the trigeminal and upper cervical systems suggests that peripheral mechanical factors may contribute to sensitization, offering complementary insights for diagnosis and adjunctive manual therapy [38].

Among second-line options, lamotrigine, a sodium channel modulator, has demonstrated encouraging efficacy. A randomized crossover trial reported pain control comparable to carbamazepine (CBZ), but with fewer central nervous system and hematological side effects [25]. Eslicarbazepine has also yielded promising results, including in patients with TN associated with multiple sclerosis, and shows a favorable tolerability profile provided that serum sodium is monitored [28]. Collectively, these findings support lamotrigine and eslicarbazepine as reasonable alternatives in patients who are refractory to or intolerant of first-line agents, consistent with expert consensus [39]. Importantly, their distinct safety advantages suggest a role not only as substitutes but also as potential early-line options in selected patient populations, highlighting the need for further head-to-head trials and long-term outcome studies.

Gabapentin (GBP), a calcium channel α2δ ligand, has emerged as a valuable therapeutic option. When combined with local anesthetic blocks, GBP enhanced pain control, quality of life, and onset of analgesia compared with monotherapy, while maintaining excellent tolerability [26]. These findings are consistent with systematic reviews and meta-analyses that highlight GBP as safer, though occasionally less potent, than sodium channel blockers, with particular benefit in continuous pain subtypes of trigeminal neuralgia [17]. Moreover, both GBP and pregabalin are especially useful in patients with prominent continuous pain or in those unable to tolerate first-line agents [40,41,42]. Taken together, these observations position α2δ ligands as possible adjuncts or alternatives within a mechanism-based framework of treatment.

Combination therapies have also shown merit, particularly in multiple sclerosis-associated trigeminal neuralgia. Regimens combining GBP with CBZ or LMT [31], or LMT with pregabalin [35], achieved meaningful pain relief while reducing the burden of adverse effects compared to high-dose monotherapy, thereby supporting rational polypharmacy approaches in refractory TN [39]. Beyond efficacy, multimodal therapy offers the advantage of targeting complementary mechanisms of action, which may allow for lower individual drug doses, improved tolerability, and better long-term adherence. These findings reinforce the potential of personalized, mechanism-based polytherapy (multimodal) to balance efficacy with safety in complex clinical scenarios. An important unresolved question, however, is whether multimodal therapy should be reserved only for refractory, atypical, or frail patients, or whether early initiation, even in patients with apparently “typical” TN, could prevent recurrence and the transition to mixed pain phenotypes. While 70–90% of patients respond initially to monotherapy, 20–30% relapse within 6–12 months due to inefficacy or poor tolerability. Exploring whether upfront multimodality could reduce these recurrences represents a relevant area for future investigation and trial design.

Levetiracetam, which targets the SV2A synaptic vesicle protein, has demonstrated modest efficacy with good tolerability and minimal interactions with other anticonvulsants [32,33]. However, evidence remains preliminary and controlled trials are needed. Topiramate, acting on AMPA-type glutamate receptors, has shown early and sustained pain relief at low doses with manageable side effects when titrated gradually [36], though data are limited. While these agents remain experimental or adjunctive [43], their distinct mechanisms of action can be tested in future studies due the potential for inclusion in multimodal treatment strategies, offering opportunities to optimize analgesia while minimizing adverse effects through combination or lower-dose regimens.

Emerging therapies like vixotrigine, a use-dependent voltage-gated sodium channel blocker, have shown favorable tolerability and potential efficacy in phase II studies [14]. However, results from large-scale phase III RCTs are still needed to confirm these findings [12,19]. These new options may expand medical treatment paradigms for TN [44].

Additional pharmacotherapies—including baclofen, topicals, and BoNT-A—have shown benefit, particularly in refractory cases or surgical preconditioning [9,45]. BoNT-A demonstrated significant pain relief in RCTs with favorable safety [11,12], and muscle relaxants like baclofen helped in up to 74% of cases [45]. These findings support a multidisciplinary, multimodal and individualized therapeutic strategy.

The findings of this review should be interpreted in light of several limitations, including heterogeneity among studies, small sample sizes, the predominance of non-randomized or open-label designs, and the exclusion of non-English publications. In addition, the overall methodological quality of the available evidence is limited, with many studies exhibiting moderate to high risk of bias, short follow-up periods, and variability in outcome definitions. These issues restrict the external validity and generalizability of the findings to broader clinical populations. Nevertheless, the review has notable strengths: it focuses on pharmacological classes, employs structured risk-of-bias assessments, and uses a class-based framework that enhances interpretability and facilitates comparison across therapies. Future studies should prioritize well-designed, larger-scale randomized controlled trials to directly compare different drug classes, drug associations, assess long-term outcomes, and evaluate combination or mechanism-based treatment strategies. Achieving this will be challenging, as illustrated by the uncertain trajectory of promising agents such as the selective Nav1.7 blocker vixotrigine [14], which showed benefit in phase II but, years later, has yet to yield definitive results in phase III trials.

## 5. Conclusions

Sodium and calcium channel modulators remain the cornerstone of pharmacological management in trigeminal neuralgia (TN), with carbamazepine and oxcarbazepine as the most effective first-line options. Lamotrigine, eslicarbazepine, and gabapentinoids provide viable alternatives for patients who are intolerant or refractory to first-line therapy. Rational multimodal/multidisciplinary combination therapies may enhance efficacy while reducing adverse effects, particularly in multiple sclerosis-associated TN, and agents such as levetiracetam and topiramate show promise as adjuncts or future therapies.

Long-term management should emphasize early pharmacologic optimization and timely consideration of surgical interventions when needed. Despite supporting evidence, the overall quality is limited by study heterogeneity, small sample sizes, the rarity of the disease, and few randomized controlled trials, underscoring the need for well-powered comparative studies with standardized outcomes. Ultimately, individualized, mechanism-based therapy tailored to clinical presentation, tolerability, and underlying etiology is essential to maximize pain control and quality of life in patients with TN.

## Figures and Tables

**Table 1:** Comparative overview of studies on Trigeminal Neuralgia pharmacological treatments and outcomes.

Ref.	Study Type Follow-Up	Country/Participants	Treatments	Response Rate/Pain Reduction/Qol	Adverse Effects
Di Stefano 2021 [29]	Non-randomized	Italy/369 (229 F/115 M), 66 years (29–89)	CBZ (600 mg) vs. OXC (1200 mg)	Initial response: 88% CBZ, 91% OXC; OXC more tolerable; late resistance in 9 patients/n.a./n.a.	AEs in 43.6% CBZ, 30.3% OXC; CBZ more severe; dizziness, somnolence, hyponatremia, rash
Domingues 2007 [36]	Case series 8 weeks	Brazil/8 (5 F/3 M), 62 years (±14)	TOP (50–100 mg/day)	6/8 improved; 3 complete remissions/n.a./n.a.	Dizziness, drowsiness, weight loss; transient
Gomez-Arguelles 2008 [27]	Non-randomized ≥12 weeks	Spain/35 (28 F/7 M), 62 years (32–84)	OXC (300–1800 mg/day, mean 774 mg)	Relief in 1–3 weeks; sustained/≥50% pain reduction in 67.5%/positive perception in 25/35	Well tolerated; mild neuro/GI effects; hyponatremia in 10 patients
Jorns 2009 [32]	Non-randomized 10 weeks	/10 (5 F/5 M), 47–81 years	LEV (3–5 g/day)	40% response/n.a./some QoL improvement	Mild AEs: dizziness, fatigue, diarrhea
Lemos 2008 [26]	RCT 1 year	Portugal/36 (20 F/16 M), 61–64 years	Group1: GBP; Group 2: Ropivacaine; Group 3: Combination	Pain reduction for all; combination most effective, lower GBP dose/better QoL	No AEs in combination; GBP alone linked to somnolence
Mitsikostas 2010 [33]	Non-randomized 20 weeks	Greece/23 (14 F/9 M), 60 years (22–78)	LEV (3–4 g/day, adjunctive)	62% crisis reduction/n.a./improved anxiety and depression	21% AEs (dizziness, rash, GI); 2 discontinued
Noro 2021 [30]	Non-randomized 24 h	Japan/20 (10 F/10 M), 68 years (32–88)	IV Fosphenytoin (750 mg)	19/20 with significant relief/Rapid relief within minutes; partial effect up to 24 h/n.a.	Mild transient dizziness in 4 patients
Sanchez-Larsen 2018 [28]	Non-randomized 7 days–78 months (mean 21 months)	Spain/18 (15 F/3 M), 65 years (28–92)	ESL (200–1200 mg)	Response in 88.9%/Pain intensity reduced (9.5 → 2.5) and frequency (70 → 0.37/week)/n.a.	61% mild/transient AEs; 4 withdrawals (severe dizziness, myoclonus, hyponatremia)
Shaikh 2011 [25]	RCT 20 weeks	Malaysia/21 (12 F/9 M), 65 years (32–84)	LMT (400 mg) vs. CBZ (1200 mg)	LMT effective in 62%; CBZ in 90%/LMT gave more complete relief in responders/n.a.	Similar safety; LMT: rash, headache, dizziness; CBZ: headache, dizziness, nausea, 2 Stevens-Johnson
Solaro 2000 [31]	Non-randomized 2 months	Italy/11 (9 F/2 M), 49–52 years	Group 1: CBZ + GBP; Group 2: LMT + GBP	Almost all achieved pain control; AEs resolved after GBP/n.a.	1 mild imbalance; overall well tolerated
Solaro 2018 [35]	Case series 3 months	Italy/5 (3 F/2 M), 39–69 years	LMT (100–300 mg) + PGB (150–225 mg)	Maintained relief/pain score 3 → 0/1 in all patients/n.a.	Initial dizziness, ataxia, malaise; well tolerated after adjustment
Zakrzewska 2002 [34]	Clinical trial 16 ± 6 years	UK/15 (11 F/4 M), 55 years (38–78)	OXC (1200 ± 600 mg/day, 4 years), ± surgery	Short-term effect of OXC may lead some patients to eventually require surgery/n.a./n.a.	OXC: mild side effects and a dose-dependent hyponatraemia.

Legend: Ref.—reference, AEs, Adverse Effects; CBZ, Carbamazepine; ESL, Eslicarbazepine; F, Female; GBP, Gabapentin; IV, Intravenous; LEV, Levetiracetam; LMT, Lamotrigine; M, Male; OXC, Oxcarbazepine; QoL, Quality of Life; PGB, Pregabalin; RCT, Randomized Clinical Trial; TOP, Topiramate; UK, United Kingdom.

**Table 2:** Summary of key findings involving the main pharmacological approach for trigeminal neuralgia.

Drug Class	Anticonvulsant	Key Findings	Efficacy
Sodium channel inhibitors	Carbamazepine	High efficacy in classical TN but limited by CNS and hematological side effects.	Moderate to High
	Oxcarbazepine	Similar efficacy to carbamazepine with better tolerability. Hyponatremia is common.	
	Lamotrigine	Effective and better tolerated alternative due to fewer CNS/hematological side effects.	Moderate
	Eslicarbazepine	Promising results, including in MS-related TN. Hyponatremia is common.	
Calcium channel inhibitors	Gabapentin	Combination with local anesthetic block (ropivacaine) enhances outcomes.	Low to Moderate
	Ropivacaine	Used as peripheral nerve blocker. Enhances pain relief when combined with GBP.	
SV2A ligand	Levetiracetam	Partial efficacy in TN with good tolerability. May be useful as adjunctive therapy.	Limited
Topiramate		Effective monotherapy in some patients. Lower doses may improve tolerability. Limited evidence.	Low to Moderate

Legend: CNS, Central Nervous System; MS, Multiple Sclerosis; SV2A, Synaptic Vesicle Protein 2A; TN, Trigeminal Neuralgia.

## Data Availability

All data generated during this study are included within the study.

## Supplementary Materials

The following supporting information can be downloaded at https://www.mdpi.com/article/10.3390/medicines13010003/s1: PRISMA 2020 checklist.

## Abbreviations

The following abbreviations have been used in this manuscript:

AMPA	α-amino-3-hydroxy-5-methyl 4-isoxazolepropionic acid
BoNT-A	Botulinum Toxin Type A
CBZ	Carbamazepine
CGRP	Calcitonin Gene-Related Peptide
ESL	Eslicarbazepine
GBP	Gabapentin
IHS	International Headache Society
LEV	Levetiracetam
LMT	Lamotrigine
MS	Multiple Sclerosis
OXC	Oxcarbazepine
PGB	Pregabaline
PRISMA	Preferred Reporting Items for Systematic Reviews and Meta-Analyses
PROSPERO	International Prospective Register of Systematic Reviews
QOL	Quality of Life
RCT	Randomized Clinical Trial
SV2A	Synaptic Vesicle Protein 2A
TN	Trigeminal Neuralgia
TOP	Topiramate
VAS	Visual Analog Scale
